# Responding Simultaneously to Flood and COVID-19 in Iran

**DOI:** 10.1017/dmp.2021.6

**Published:** 2021-01-11

**Authors:** Farin Fatemi, Shandiz Moslehi

**Affiliations:** 1 Research Center for Health Sciences and Technologies, Semnan University of Medical Sciences, Semnan, Iran; 2 Health Management and Economics Research Center, Iran University of Medical Sciences, Tehran, Iran; 3 Department of Health in Disasters and Emergencies, School of Health Management and Information Sciences, Iran University of Medical Sciences, Tehran, Iran

**Keywords:** Floods, climate change, health impact, Iran, COVID-19

Global climate change has increased the number and intensity of extreme climate events such as floods, heat or cold waves, and drought.^1^ Based on this fact, Iran has experienced massive floods during the past 2 decades; the most disastrous ones happened on March 2019 with 2 million affected people and more than 4 billion dollars in costs.^2^ Iran was successful in controlling the current epidemics of viral infections after the flood in March 2019 because the health system could establish a rapid surveillance system to allow early detection and isolation of the patients in the field hospitals in the flood-stricken areas.^2^

Due to weather predictions and flood warnings by the Iranian Meteorology Organization (IMO) in winter and spring 2020-2021 for 15 provinces, coinciding with the coronavirus disease 2019 (COVID-19) crisis in Iran, flood management has become a more complex issue. Iran, like many other countries, has been severely affected by the coronavirus pandemic, and the health-care system is being overwhelmed after 9 mo of fighting against COVID-19. However, addressing this issue scientifically, literature reviews and expert panels have recommended the most important measures applicable to better management of floods during the presence of COVID-19 in Iran^3-5^:

- Enhancing whole-of-society coordination mechanisms to support preparedness including the health, Red Crescent, transport, travel, security, and other first responders to crisis
- Establishing emergency camps or temporary settlements for displaced populations who should leave their homes
- Early detection of the patients of COVID-19 in the displaced population and isolating sick individuals by establishing a surveillance system in emergency camps and temporary settlements
- Control of critical points for managing Coronavirus-infected waste in health-care centers or field hospitals
- Meeting the flood-stricken people’s emergency health needs by paying special attention to vulnerable people, particularly the elderly, women, and children
- Intensive training and reminders of the public health measures, including hand hygiene, respiratory etiquette, and social distancing, by health-care providers in the provinces that have received the flood warnings
- Supply of medicine, determination of distribution locations and how patients access outpatient drugs, and training of delivery and drug distribution personnel
- Weekly distribution of adequate quantities of hygiene items such as masks, soap, waterless antiseptic agents (ie, alcohol-based solutions) for disinfecting hands and surfaces among flood-stricken people
- Control shared bathrooms, clean surfaces with water and detergents, and use appropriately designed toilets to prevent the contamination of groundwater resources
- Access to psychological consults and supports for flood-affected people, particularly for households that lost member(s) due to COVID-19

It is recommended that policy-makers and health-care managers in flood prone areas focus on the above measures before and during the occurrence of floods. It is important for policy-makers to encourage stakeholders to manage the floods and COVID-19 using the managerial considerations that have been suggested in this study.
